# Geographic disparities and temporal changes of diabetes prevalence and diabetes self-management education program participation in Florida

**DOI:** 10.1371/journal.pone.0254579

**Published:** 2021-07-16

**Authors:** Md Marufuzzaman Khan, Shamarial Roberson, Keshia Reid, Melissa Jordan, Agricola Odoi

**Affiliations:** 1 Department of Public Health, College of Education, Health, and Human Sciences, The University of Tennessee, Knoxville, Tennessee, United States of America; 2 Florida Department of Health, Tallahassee, Florida, United States of America; 3 Department of Biomedical and Diagnostic Sciences, College of Veterinary Medicine, The University of Tennessee, Knoxville, Tennessee, United States of America; Shahjalal University of Science and Technology (SUST), BANGLADESH

## Abstract

**Background:**

Although Diabetes Self-Management Education (DSME) programs are recommended to help reduce the burden of diabetes and diabetes-related complications, Florida is one of the states with the lowest DSME participation rates. Moreover, there is evidence of geographic disparities of not only DSME participation rates but the burden of diabetes as well. Understanding these disparities is critical for guiding control programs geared at improving participation rates and diabetes outcomes. Therefore, the objectives of this study were to: (a) investigate geographic disparities of diabetes prevalence and DSME participation rates; and (b) identify predictors of the observed disparities in DSME participation rates.

**Methods:**

Behavioral Risk Factor Surveillance System (BRFSS) data for 2007 and 2010 were obtained from the Florida Department of Health. Age-adjusted diabetes prevalence and DSME participation rates were computed at the county level and their geographic distributions visualized using choropleth maps. Significant changes in diabetes prevalence and DSME participation rates between 2007 and 2010 were assessed and counties showing significant changes were mapped. Clusters of high diabetes prevalence before and after adjusting for common risk factors and DSME participation rates were identified, using Tango’s flexible spatial scan statistics, and their geographic distribution displayed in maps. Determinants of the geographic distribution of DSME participation rates and predictors of the identified high rate clusters were identified using ordinary least squares and logistic regression models, respectively.

**Results:**

County level age-adjusted diabetes prevalence varied from 4.7% to 17.8% while DSME participation rates varied from 26.6% to 81.2%. There were significant (p≤0.05) increases in both overall age-adjusted diabetes prevalence and DSME participation rates from 2007 to 2010 with diabetes prevalence increasing from 7.7% in 2007 to 8.6% in 2010 while DSME participation rates increased from 51.4% in 2007 to 55.1% in 2010. Generally, DSME participation rates decreased in rural areas while they increased in urban areas. High prevalence clusters of diabetes (both adjusted and unadjusted) were identified in northern and central Florida, while clusters of high DSME participation rates were identified in central Florida. Rural counties and those with high proportion of Hispanics tended to have low DSME participation rates.

**Conclusions:**

The findings confirm that geographic disparities in both diabetes prevalence and DSME participation rates exist. Specific attention is required to address these disparities especially in areas that have high diabetes prevalence but low DSME participation rates. Study findings are useful for guiding resource allocation geared at reducing disparities and improving diabetes outcomes.

## Introduction

Diabetes is the seventh leading cause of death in the United States (US) and is characterized by Fasting Plasma Glucose (FPG) levels of ≥126 mg/dl while the FPG levels for prediabetes is 100-<126 mg/dl [1]. Over the last 20 years, the number of diabetic patients in the US has doubled and is projected to double or triple again by 2050 [2]. The economic burden of the condition is quite significant as evidenced by the fact that the average healthcare expenditure of a diabetic patient is 2.3 times higher than that of a non-diabetic. The total estimated cost of the condition in the US, including direct (treatment) and indirect (reduced productivity) costs, is $327 billion [3]. The increasing burden of diabetes observed in the US has been reported in Florida as well. For example, the prevalence of diabetes among Florida adults increased from 5.2% in 1995 to 12.6% in 2018 [4,5]. Moreover, almost 7.3% of adults in Florida have prediabetes [6]. Individuals with prediabetes have a higher risk of developing diabetes compared to those that do not have the condition [7]. On average, Florida spends $24.3 billion each year on diabetes and prediabetes [8,9].

The National Diabetes Educational Program (NDEP) was jointly launched by the Department of Health and Human Services (HHS), National Institutes of Health (NIH), and Centers for Disease Control and Prevention (CDC) to provide educational and preventive programs intended to reduce the risks and complications of diabetes [10]. The Diabetes Self-management Education (DSME) is one of those educational programs developed to minimize development of diabetes related complications and improve clinical outcomes as well as quality of life of diabetic patients [11,12]. Unfortunately, less than 7% of newly diagnosed diabetic patients in the US participate in this program within the first year of diagnosis [13]. Moreover, only about 54.4% of diabetics in the US attended DSME classes in 2015. This was the lowest participation rate among all the existing CDC recommended preventive measures for diabetes. In Florida, the rate was even lower (45.2%) than the national average [14].

There is evidence of geographic and sociodemographic disparities related to diabetes prevalence [15], DSME program availability [16], emergency department visits [17], and hospitalizations in the US [18]. However, disparities in DSME participation have not been investigated. Identifying these disparities is important for guiding health planning and service provision to minimize/eliminate the disparities, reduce the burden of diabetes and diabetes related complications and improve population health. Therefore, the objectives of this study were to: (a) investigate geographic disparities and temporal changes in diabetes prevalence and DSME program participation rates in Florida between 2007 and 2010; (b) identify predictors of the geographic disparities in DSME participation rates in Florida. Study findings will be useful for guiding prevention and control programs and policy.

## Materials & methods

### Study area

**Fig 1:**
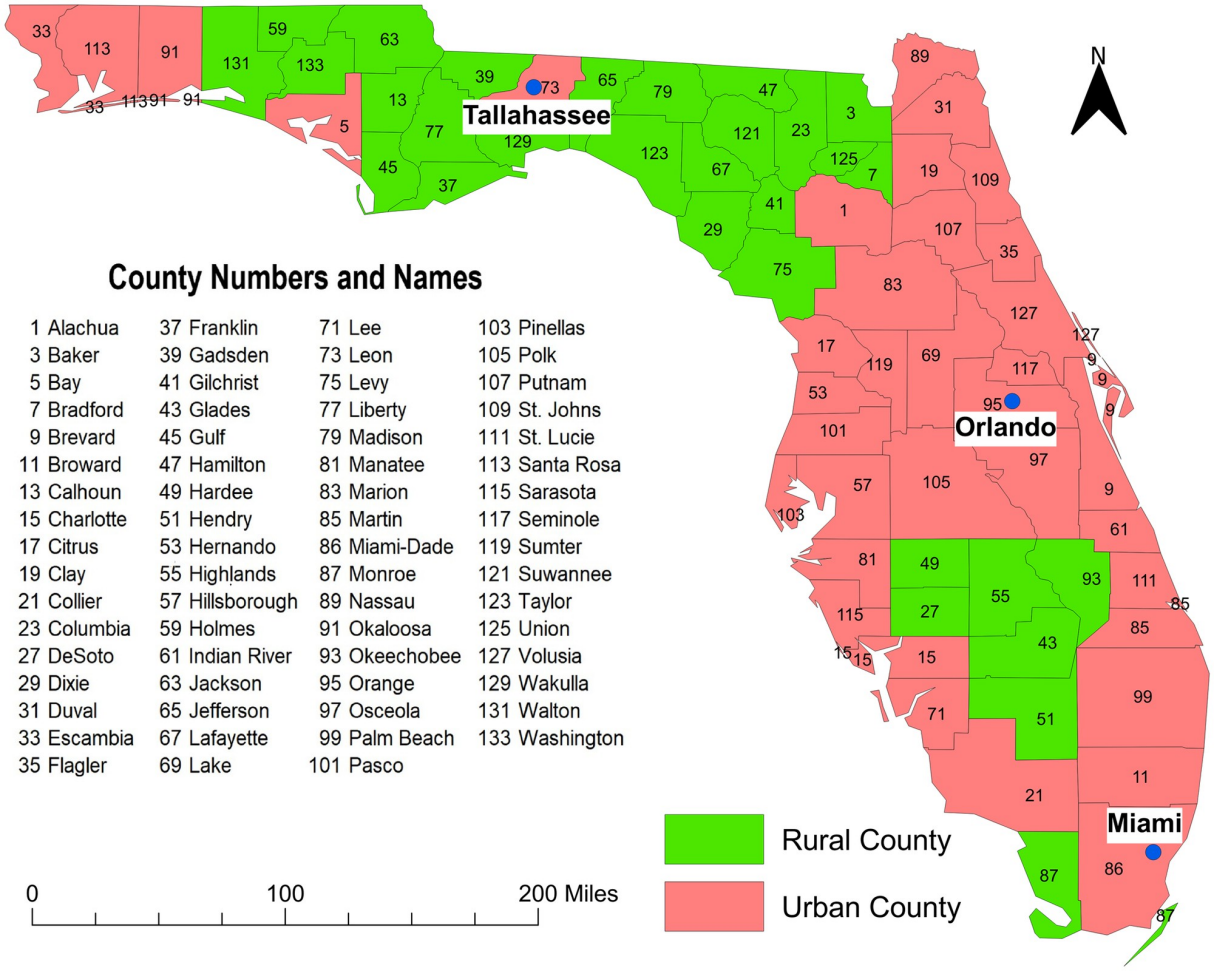
Map of Florida showing geographic distribution of urban and rural counties. Figure was created using the Free and Open Source Geographic Information System (GIS) software, QGIS. The basemaps used to create the maps were downloaded from the US Census Bureau Website: https://www.census.gov/geographies/mapping-files/time-series/geo/tiger-line-file.2010.html. The source of the data displayed in the map is Florida Department of Health. Data are available at: http://www.floridahealth.gov/programs-and-services/community-health/rural-health/_documents/rual-counties-2000-2010.pdf.

This retrospective ecological study was performed in the state of Florida and included data from the years 2007 and 2010. Florida has 67 counties many of which are located in the diabetes belt which is an area of the US having a higher prevalence of diabetes (11.7%) than the rest of the country (8.5%) [19]. As of 2018, Florida was the most populous state in the southeastern US with approximately 20.9 million people. It has the second-highest number of the elderly (≥65 years old) population in the US [20,21]. The age distribution of the population is 0–19 year-olds (22.3%), 20–34 year-olds (19.2%), 35–44 year-olds (12.0%), 45–54 year-olds (13.2%), 55–64 year-olds (13.3%) and ≥65 year-olds (20%). Approximately 51% of the population is female. The majority (77.4%) of the population is white, 16.9% is black while all other races comprise 5.7% of the population. By ethnicity, 25.7% of the population is Hispanic-Latino while the rest is non-Hispanic [21]. The state has both urban and rural areas with Miami-Dade being the most urban and populous county (2,804,160 residents) and Lafayette county being the most rural and least populous (8,367 residents) [21] (Fig 1).

### Data sources

Data on diabetes and DSME, for the years 2007 and 2010, were extracted from the Behavioral Risk Factor Surveillance System (BRFSS) datasets that were obtained from the Florida Department of Health. The 2007 and 2010 are the latest available DSME participation data currently available because the Florida 2016 and 2019 BRFSS questionnaire did not include questions on DSME participation. The BRFSS collects data from adults 18 years of age or older. Diabetes status was determined based on the respondent’s report of having been told by a doctor that they had diabetes not related to pregnancy. The survey did not make a distinction between type 1 and 2 diabetes. The DSME participation was determined using diabetic patients’ responses to the question regarding if they had ever participated in a DSME program. Additional data extracted from the BRFSS datasets included respondent’s county of residence, age, gender, race, marital status, educational attainment, healthcare accessibility, body mass index (BMI), exercise, smoking, and drinking habits. Rural counties were identified based on the classification available at the Florida Department of Health website [21]. This classification is based on the population density of the county. Thus, rural counties were defined as those with population density of ≤100 persons per square mile. County-level proportion of the rural population, unemployed population, and median income were obtained from the County Health Rankings and Roadmap (CHRR) website [22]. County-level proportion of population below the federal poverty level was obtained from the American Community Survey (ACS) 5-years estimates [23]. Cartographic boundary file for county-level geographic analyses was downloaded from the United States Census Bureau TIGER Geodatabase [24].

### Data preparation and visualization

The BRFSS data were aggregated to the county level using SAS 9.4 [25]. Since these data were collected using a complex survey design, a weight variable (created by the US Centers for Disease Control and Prevention [CDC]) was used for all individual level analyses [26,27]. Thus, all county-level estimates/variables, derived from individual level survey responses, were computed using the weight variable to ensure that the estimates were generalizable to all Florida adults. Therefore, computation of county-level percentages/frequencies involved using SURVEYFREQ procedure of SAS and specifying the strata variable (_STSTR), cluster variable (_PSU) as well as a sampling weight variable (_FINALWTSTAT). County level variables included in the study were: percentage of population in each of the following variable categories: DSME participation (Yes/No), BMI categories (<25 [Neither overweight nor obese], 25–30 [Overweight], and >30 [Obese]); Education (≤High school education, Some college education, College education); Participation in any physical activities (Yes/No); Ever smoked (Yes/No); Heavy alcohol consumption defined as adult men having more than two drinks per day and adult women having more than one drink per day (Yes/No); Race and ethnicity (non-Hispanic White, non-Hispanic Black, Hispanic, and Others); Gender (Male, Female); Health status (Good, Fair or poor); Used insulin for controlling diabetes (Yes/No); Had limited activity due to physical, mental or emotional problems (Yes/No); Had retinopathy as a complication of diabetes (Yes/No); Availability of primary health care provider (Yes/No); Marital status (Married, Divorced or widowed or separated, Never married); Age (Population of 18–44 years, >44–64 years, and ≥65 years); population ≥25 years with a college degree; population living below the federal poverty level. Direct age-standardized county-level diabetes prevalence was calculated using the 2000 population of Florida as the standard [28]. Since the BRFSS data only contains data from respondents aged ≥18 years, county-level age-adjusted diabetes prevalence were computed using the following age categories: 18–44, >44–64 and ≥64 years [28].

### Descriptive analysis

All descriptive analyses were performed in SAS 9.4 [25]. Normality of continuous county-level variables were assessed using Q-Q plots and Shapiro-Wilk tests. The Shapiro-Wilks test was used because it has been shown to have high power compared to other common methods such as Kolmogorov-Smirnov and Anderson-Darling [29]. Mean and confidence intervals were used to summarize normally distributed variables while median and lower-upper quartiles were used for variables showing deviations from normality.

### 2007 to 2010 comparisons

One-tailed tests of equality of proportions were performed to identify significant increases or decreases in diabetes prevalence and DSME participation rates between 2007 and 2010 using STATA [30] command ‘prtest’. Simes method was used to adjust for multiple comparisons [31].

### Clusters of high diabetes prevalence and high DSME participation rates

A Poisson model, implemented in SAS 9.4, was used to adjust diabetes prevalence for the following known risk factors: age, gender, race/ethnicity, and BMI [32,33]. Tango’s flexible spatial scan statistics (FSSS), implemented in FlexScan [34], was then used to identify circular and irregularly shaped spatial clusters of both unadjusted and adjusted high diabetes prevalence. Tango’s FSSS was also used to identify clusters of high DSME participation rates. Poisson probability models with restricted log likelihood (LLR) ratio (specifying alpha of 0.2) and maximum cluster size of 15 counties were specified to preclude potential inclusion of counties with non-elevated prevalence proportions or participation rates. To identify statistically significant clusters, 999 Monte Carlo replications were used specifying a critical p-value of 0.05. For each outcome, the significant cluster with the largest value of restricted LLR was identified as the primary cluster. The rest of the significant clusters were secondary clusters and were ranked based on their restricted LLR values. Clusters with prevalence ratios (PR) or participation rate ratios (PRR) less than 1.2 were not reported to avoid reporting very low prevalence or low rate clusters.

### Predictors of geographic distribution of DSME participation rates

To investigate the predictors of county-level DSME participation rates, a multivariable ordinary least squares regression model was built using SAS 9.4 [35] in two steps. The outcome variable was specified as county-level DSME participation rates. The 1^st^ step of model building involved univariable assessments to identify potential predictors of DSME participation rates. Variables considered for potential univariable association with participation rates are listed in Table 1. Only potential predictor variables significant at a liberal p≤0.15 were considered for building the multivariable model in the 2^nd^ step.

**Table 1:** Summary statistics of variables considered as potential predictors of county-level Diabetes Self-management Education (DSME) program participation and its hotspots in Florida, 2010.

Predictor variable	Mean	SD^1^	Median	Minimum	Maximum	IQR^2^
Proportion of having normal and less than normal weight	0.333	0.061	0.336	0.180	0.460	0.071
Proportion of having overweight*	0.367	0.042	0.710	0.246	0.474	0.043
Proportion of being obese	0.300	0.069	0.293	0.173	0.497	0.079
Proportion of smokers*	0.507	0.067	0.519	0.333	0.664	0.102
Proportion of heavy drinkers	0.055	0.019	0.054	0.018	0.104	0.027
Proportion of doing exercise	0.734	0.054	0.736	0.599	0.856	0.071
Proportion of having overall good health*	0.795	0.057	0.797	0.633	0.889	0.070
Proportion of having overall poor health*	0.205	0.057	0.203	0.111	0.367	0.070
Proportion of having limited activity due to health problems*	0.264	0.046	0.261	0.184	0.454	0.061
Proportion of having high school or less education	0.438	0.122	0.439	0.200	0.679	0.201
Proportion of having some college education	0.282	0.045	0.282	0.176	0.403	0.056
Proportion of having college education*	0.280	0.108	0.266	0.118	0.519	0.175
Proportion of being white, non-Hispanic*	0.790	0.110	0.804	0.261	0.921	0.118
Proportion of being black, non-Hispanic*	0.083	0.071	0.061	0.018	0.445	0.079
Proportion of being other, non-Hispanic*	0.045	0.024	0.039	0.006	0.115	0.031
Proportion of being Hispanic*	0.082	0.077	0.068	0.006	0.537	0.069
Proportion of being married	0.632	0.053	0.638	0.509	0.760	0.077
Proportion of being divorced/widowed/separated*	0.222	0.038	0.221	0.130	0.372	0.046
Proportion of being never married	0.146	0.047	0.139	0.057	0.269	0.064
Proportion male*	0.518	0.052	0.489	0.468	0.708	0.062
Proportion female*	0.482	0.052	0.511	0.292	0.532	0.062
Proportion with age 18 to 44 years	0.381	0.087	0.386	0.183	0.575	0.125
Proportion with age 45 to 64 years*	0.394	0.075	0.382	0.288	0.555	0.132
Proportion with age equal or greater than 65 years*	0.224	0.069	0.204	0.102	0.397	0.093
Median household income (in $10,000)*	4.181	0.691	4.118	3.097	6.084	1.004
Proportion of being unemployed	0.110	0.019	0.114	0.082	0.156	0.027
Proportion of having diabetes	0.096	0.026	0.095	0.047	0.178	0.036
Proportion that take insulin	0.302	0.086	0.293	0.128	0.526	0.112
Proportion that have diabetic complications (retinopathy)*	0.201	0.065	0.188	0.094	0.467	0.073
Proportion that have regular healthcare provider access*	0.799	0.055	0.805	0.560	0.902	0.070
Proportion of rural population*	0.375	0.323	0.238	0.000	1.000	0.590
Proportion that have insurance coverage	0.741	0.079	0.753	0.482	0.911	0.116
Proportion with age ≥25 years with a college degree*	0.294	0.112	0.286	0.126	0.553	0.182
Proportion below the federal poverty level*	0.177	0.051	0.165	0.098	0.297	0.082

^1^Standard deviation.

^2^Interquartile range.

*Non-normally distributed variables.

Spearman’s rank correlation coefficient was used to identify highly correlated (r ≥ 0.7) variables. Only one of a pair of highly correlated variables was retained for assessment in the multivariable model. The decision regarding which of a pair of highly correlated variables to retain was based on biological and statistical considerations. The 2^nd^ step involved building a multivariable ordinary least squares regression model using a manual backwards elimination approach using a critical p-value of ≤0.05. Confounding was assessed using change in regression coefficients of variables in the model when it was run with and without a suspected confounder. If removal of a suspected confounding variable resulted in a change of 20% or more of any of the other variables in the model, then the variable was kept in the model as a confounder regardless of its statistical significance. Biologically meaningful two-way interaction terms of variables in the final main effects model were assessed with the aim of keeping significant ones. Multicollinearity was assessed using both variance inflation factor (VIF) and multicollinearity condition number. Values of VIF>10 or multicollinearity condition number >20 were considered indicative of multicollinearity. Heteroskedasticity and normality of residuals were assessed using White and Jarque-Bera tests, respectively. Robust Lagrange Multiplier (LM) tests, employing inverse distance spatial weights, were used to assess for spatial dependence of residuals.

### Predictors of clusters of high DSME participation rates

To investigate the predictors of clusters of high DSME participation rates, logistic regression model was built in SAS 9.4 [25]. The outcome variable for the logistic regression was a binary variable (Yes/No) indicating whether or not a county belonged to a high DSME participation rate cluster. The logistic model was also built in two steps as described above except in this case the outcome variable was dichotomous (Yes/No) representing whether a county was part of a high DSME participation rate cluster or not. Goodness-of-fit of the logistic model and spatial dependence were assessed using Hosmer-Lemeshow test and Moran’s I using inverse distance spatial weights, respectively [36,37].

### Cartographic displays

All cartographic displays were generated using the Free and Open Source Geographic Information System (GIS) software, QGIS [38]. The prevalence estimates of diabetes, DSME participation rates, and its predictors, as well as significant spatial clusters, were displayed on maps. Jenk’s optimization classification scheme was used to determine critical intervals for choropleth maps. In addition, statistically significant changes in county-level estimation of diabetes prevalence and DSME participation rates between 2007 and 2010 were displayed using manual intervals classification scheme.

### Ethics approval

This study was reviewed by the University of Tennessee Institutional Review Board (Number: UTK IRB-20-05707-XM) and determined to be eligible for exempt review under 45 CFR 46.101. Category 4: Secondary research for which consent is not required. The study used anonymized secondary data provided to the investigators in such a manner that the identity of the human subjects cannot be ascertained directly or through identifiers linked to the subjects. The investigators did not contact the subjects and did not re-identify subjects.

## Results

### Spatial distribution

**Fig 2:**
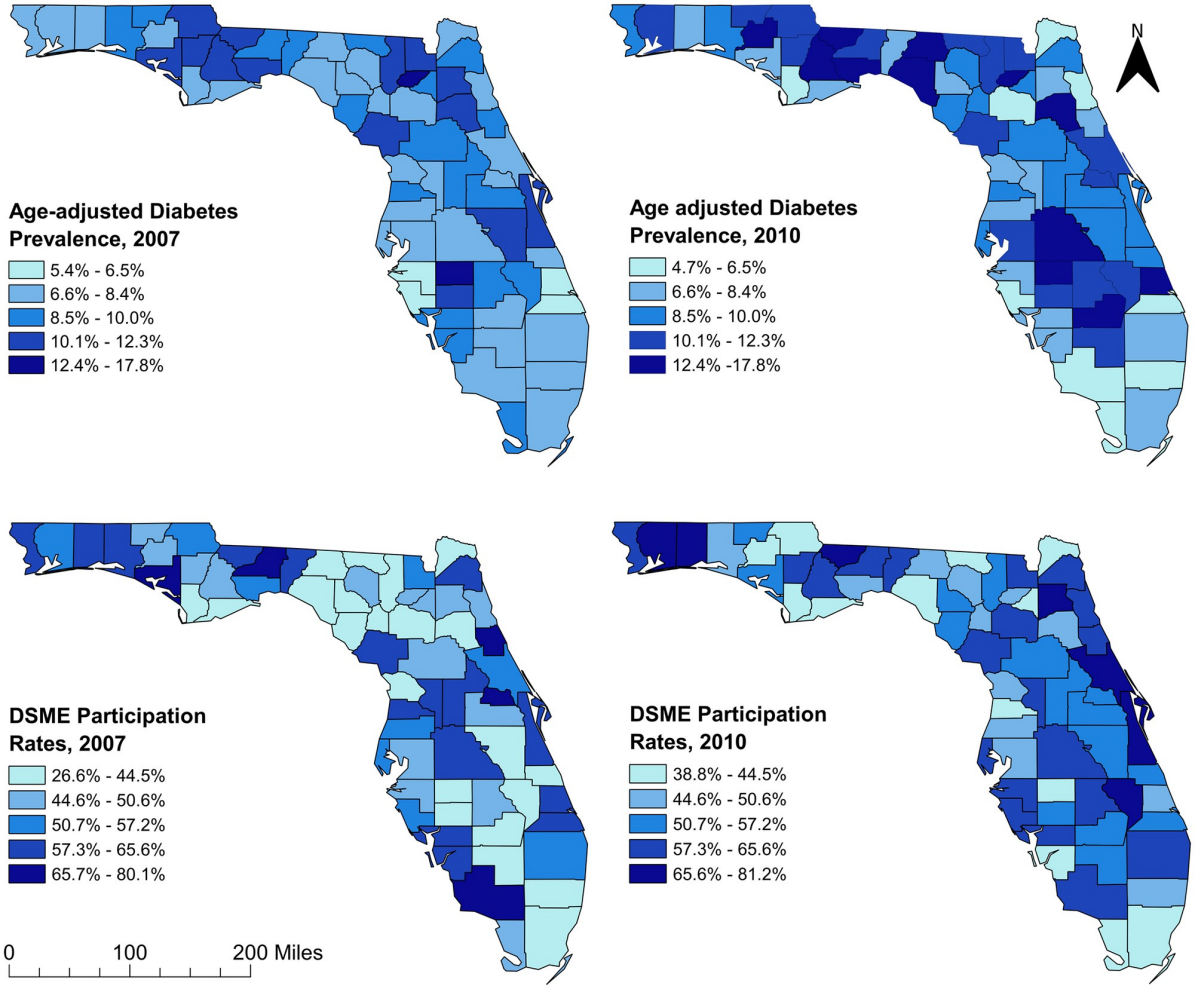
Age-adjusted county level diabetes prevalence and diabetes self-management education program participation rates in Florida, 2007–2010. Figure was created using the Free and Open Source Geographic Information System (GIS) software, QGIS. The basemaps used to create the maps were downloaded from the US Census Bureau Website: https://www.census.gov/geographies/mapping-files/time-series/geo/tiger-line-file.2010.html. The diabetes prevalence and diabetes self-management education (DSME) participation rate data were obtained from Florida Department of Health Website. These data are available at: http://www.flhealthcharts.com/charts/Brfss/DataViewer.aspx?bid=21 (prevalence data) and http://www.flhealthcharts.com/charts/Brfss/DataViewer.aspx?bid=51 (DSME data).

The age-adjusted diabetes prevalence varied across counties in Florida ranging from 4.7% to 17.8% (Fig 2). In 2007, 16 counties in the panhandle, north-central and mid-Florida had diabetes prevalence greater than 10% while almost half (28) of the counties in those same areas had prevalence greater than 10% in 2010. Most of the counties with high prevalence were located in rural areas (Figs 1 and 2). On the other hand, diabetes prevalence of several urban counties in southern and northeast Florida were lower in 2010 compared to 2007.

Diabetes self-management Education program participation rates also varied across counties in Florida ranging from 26.6% to 81.2% (Fig 2). Between 2007 and 2010, DSME participation rates decreased in several counties of the central panhandle area of northern Florida while they increased in the entire north-central to mid-Florida. Overall, between 2007 and 2010, DSME participation rates decreased in rural areas while they increased in urban areas (Figs 1 and 2).

### Changes in diabetes prevalence and DSME participation rates, 2007–2010

**Fig 3:**
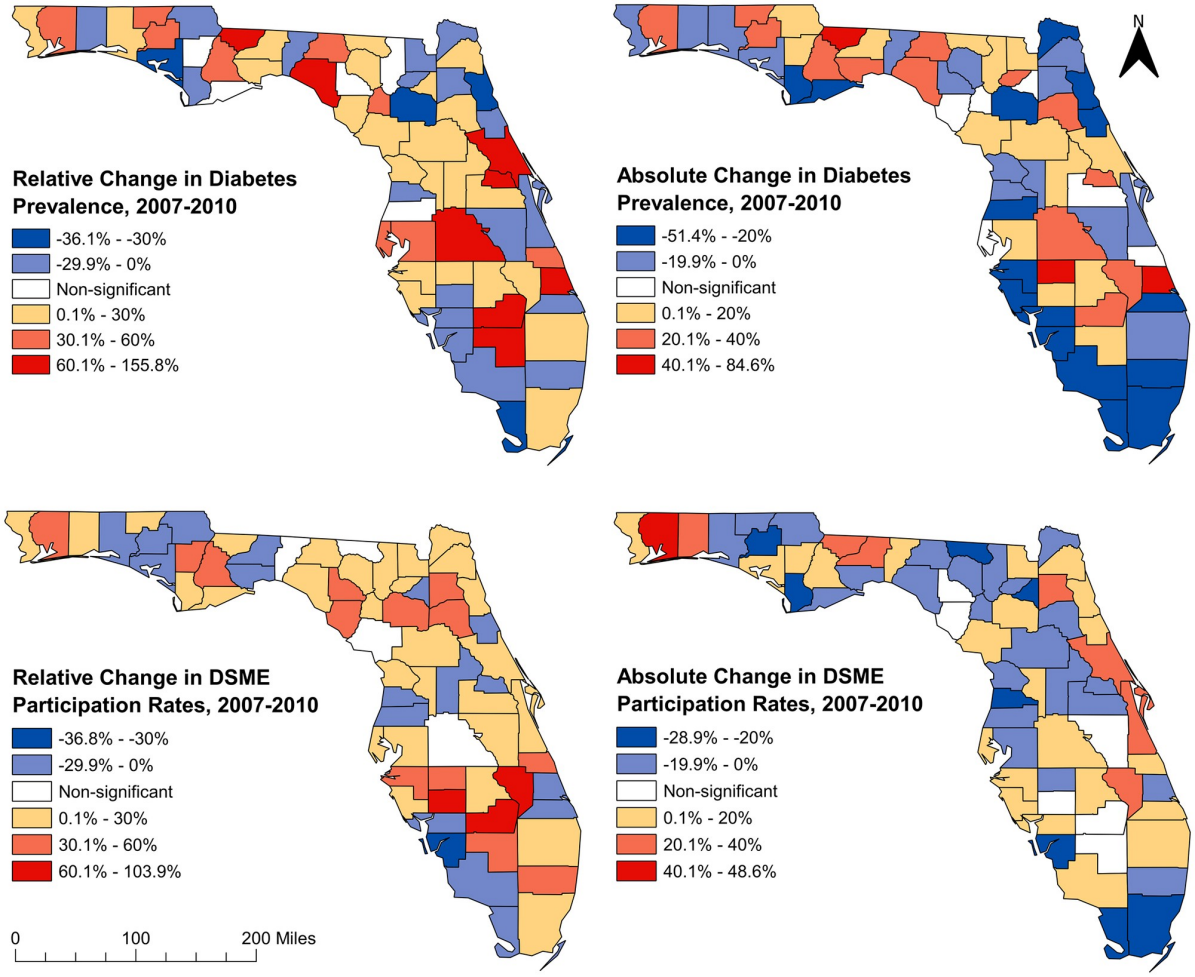
Relative and absolute changes of diabetes prevalence and diabetes self-management education program participation rates in Florida between 2007 and 2010. Figure was created using the Free and Open Source Geographic Information System (GIS) software, QGIS. The basemaps used to create the maps were downloaded from the US Census Bureau Website: https://www.census.gov/geographies/mapping-files/time-series/geo/tiger-line-file.2010.html. The diabetes prevalence and diabetes self-management education (DSME) participation rate data were obtained from Florida Department of Health Website. These data are available at: http://www.flhealthcharts.com/charts/Brfss/DataViewer.aspx?bid=21 (prevalence data) and http://www.flhealthcharts.com/charts/Brfss/DataViewer.aspx?bid=51 (DSME data).

There was a statistically significant (p<0.001) increase in the overall state-wide age-adjusted diabetes prevalence from 7.7% in 2007 to 8.6% in 2010. Sixty-two of the 67 counties had significant changes (either increases or decreases) in diabetes prevalence (Fig 3). The five counties that did not have significant changes in diabetes prevalence over the time period were Calhoun, Franklin, Lafayette, Columbia, and Pasco counties (Figs 1 and 3). Statistically significant (p<0.05) decreases in diabetes prevalence were observed in 35.5% (22/62) of the counties that had significant changes, while significant (p<0.05) increases were seen in 64.5% (40/62) of these counties. The largest increase in diabetes prevalence (14.1%, a relative increase of 155.8%) was observed in St. Lucie (south-east coastal county) whereas the largest decrease (5.1%, a relative decrease of 36.1%) was observed in St. Johns (north-east coastal county) (Figs 1 and 3).

Significant (p<0.001) state-wide changes in DSME participation rates were also observed in almost all the counties with the exception of Jefferson, Hamilton, Levy, and Polk counties (Figs 1 and 3). Overall, there was a statistically significant (p<0.001) increase in DSME participation rates from 51.4% in 2007 to 55.1% in 2010. Lee county had the largest significant decrease (39.6%, a relative decrease of 36.8%), while Glades county had the largest increase (54.3%, a relative increase of 103.9%). Of the counties that had significant changes in DSME participation rates, 28.6% (18/63) had significant decreases while 71.4% (45/63) had increases. It is worth noting that seven counties (Walton, Washington, Leon, Wakulla, Lake, Seminole, and St. Lucie) had significant increases in diabetes prevalence, but significant decreases in DSME participation (Figs 1 and 3). It was concerning to note that although St. Lucie had the largest increase (155.8%) in diabetes prevalence, it had 20.9% decrease in DSME participation rate. Moreover, a similar pattern was also observed in Leon county where the state administrative capital is located (Figs 1 and 3).

### Clusters of diabetes prevalence and DSME participation rates

#### a) Unadjusted high-prevalence diabetes clusters

**Fig 4:**
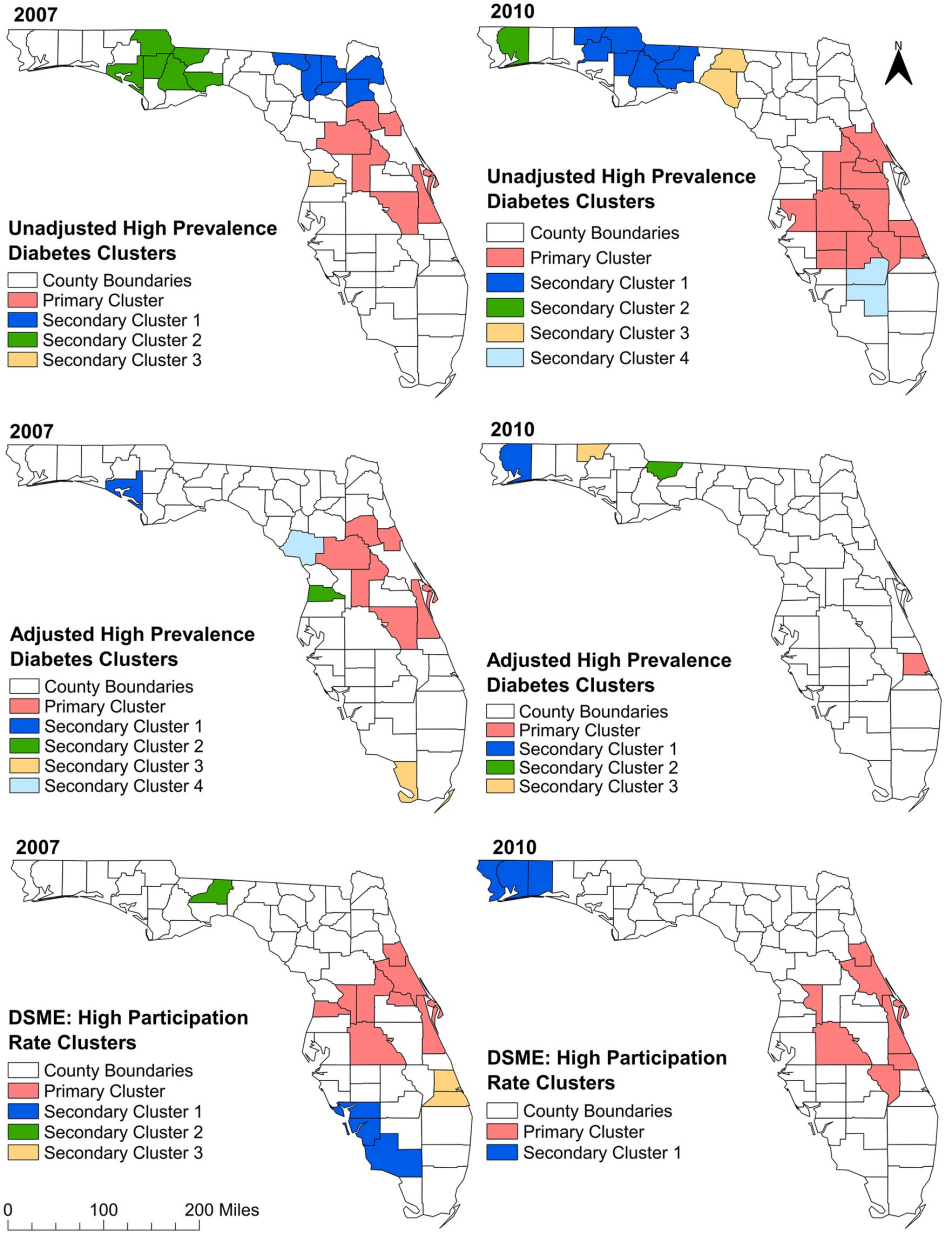
Clusters of high diabetes prevalence and high diabetes self-management education program participation rates identified in Florida using Tango’s flexible spatial scan statistics, 2007 and 2010. Figure was created using the Free and Open Source Geographic Information System (GIS) software, QGIS. The basemaps used to create the maps were downloaded from the US Census Bureau Website: https://www.census.gov/geographies/mapping-files/time-series/geo/tiger-line-file.2010.html. The diabetes prevalence and diabetes self-management education (DSME) participation rate data were obtained from Florida Department of Health Website. These data are available at: http://www.flhealthcharts.com/charts/Brfss/DataViewer.aspx?bid=21 (prevalence data) and http://www.flhealthcharts.com/charts/Brfss/DataViewer.aspx?bid=51 (DSME data).

Consistent with the increase in diabetes prevalence observed in northern and mid-Florida rural counties (Fig 3), significant high-prevalence diabetes spatial clusters were identified in these areas (Table 2 and Fig 4). There were increases in both the numbers of counties involved in the clusters and sizes of the population affected between 2007 and 2010 (Table 2 and Fig 4). A total of 4 and 5 significant spatial high-prevalence diabetes clusters were detected in 2007 and 2010, respectively. In 2007, three similar sized clusters (each containing 6 counties) of high diabetes prevalence were detected in northern and central Florida (Table 2 and Fig 4). The primary cluster in 2007 included only urban counties of central Florida while a secondary cluster included rural counties of central panhandle excluding Leon County, where the state capital is located. It is worth mentioning that this secondary cluster had the highest prevalence ratio (PR = 1.45: p = 0.001) in 2007. Another secondary cluster (Secondary Cluster 1) that was identified in 2007 had 23% higher diabetes prevalence than the state average and included several counties (Hamilton, Columbia, Union, Baker, Clay, Duval), at the urban-rural interface in the north, that were not part of any cluster in 2010 (Figs 1 and 4). Interestingly, only two of the counties (Lake and Osceola) that were part of the primary cluster in 2007 were also part of a cluster (Primary Cluster) in 2010. The primary cluster in 2010 was much larger (included 13 counties) and was located in mid-Florida (Table 2 and Fig 4). Moreover, the northern secondary cluster identified in 2007 expanded in 2010 and included Leon county, an urban county where the state capital is located. In 2010, the cluster that had the highest diabetes prevalence ratio (PR = 1.46; p = 0.001) included two rural counties of north Florida, Madison and Taylor counties (Figs 1 and 4). Both counties were not even a part of any cluster in 2007.

**Table 2:** Purely spatial unadjusted high-prevalence diabetes clusters in Florida, 2007 and 2010.

Cluster	Population	Observed cases	Counties included^2^	No of Counties included	PR^1^	*p*-value
			**2007**			
Primary	1,239,259	123,826	9, 35, 69, 83, 97, 107	6	1.29	0.001
Secondary 1	857,484	82,033	3, 19, 23, 31, 47, 125	6	1.23	0.001
Secondary 2	243,828	27,400	5, 13, 39, 63, 77, 129	6	1.45	0.001
Secondary 3	126,678	11,846	53	1	1.21	0.001
			**2010**			
Primary	3,900,382	427,501	27, 49, 55, 57, 61, 69, 93, 95, 97, 105, 111, 117, 127	13	1.26	0.001
Secondary 1	374,678	44,435	13, 39, 59, 63, 73, 77, 129, 133	8	1.36	0.001
Secondary 2	116,875	13,885	113	1	1.37	0.001
Secondary 3	33,972	4,320	79, 123	2	1.46	0.001
Secondary 4	38,710	4,464	43, 51	2	1.33	0.001

^1^Prevalence ratio.

^2^[3 = Baker 5 = Bay 9 = Brevard 13 = Calhoun 19 = Clay 23 = Columbia 27 = Desoto 31 = Duval 35 = Flagler 39 = Gadsden 43 = Glades 47 = Hamilton 49 = Hardee 51 = Hendry 53 = Hernando 55 = Highlands 57 = Hillsborough 59 = Holmes 61 = Indian River 63 = Jackson 69 = Lake 73 = Leon 77 = Liberty 79 = Madison 83 = Marion 93 = Okeechobee 95 = Orange 97 = Osceola 105 = Polk 107 = Putnam 111 = St. Lucie 113 = Santa Rosa 117 = Seminole 123 = Taylor 125 = Union 127 = Volusia 129 = Wakulla].

Some high-prevalence clusters were persistent over the study period. These included counties in northern Florida (Jackson, Calhoun, Gadsden, Liberty, Wakulla counties) and mid-Florida (Lake, Osceola counties) (Figs 1 and 4). All of them were rural counties, except Lake and Osceola. Some counties transitioned from non-clusters to statistically significant high-prevalence clusters and these were mostly located in mid-Florida. However, clusters located in north-central Florida had the opposite trend of transitioning from significant clusters to non-cluster counties. Ten counties (both urban and rural) were clusters in 2007 but became non-clusters in 2010. With the exception of Indian River, St Luis and Volusia counties, the majority of the urban counties (from both east and west coasts) and spanning from north to south, transitioned to or remained as non-cluster counties in 2010 (Figs 1 and 4). Brevard county that was part of the primary cluster in 2007 was not part of a cluster in 2010 (Table 2; Figs 1 and 4).

#### b) Risk-factor adjusted high-prevalence diabetes clusters

The number of counties that were part of high-prevalence clusters in both 2007 and 2010 were lower for adjusted than unadjusted prevalence clusters (Tables 2 and 3; Fig 4). Several of the northern counties (in both 2007 and 2010) that were part of the unadjusted prevalence clusters were not in clusters after risk-factor adjustment (Fig 4). In a nutshell, although significant clusters were still identified after risk adjustment, the number of affected counties significantly reduced after risk adjustment implying that the risk factors explained the high prevalence in several counties that were part of the unadjusted clusters.

**Table 3:** Purely spatial risk-factor adjusted high-prevalence diabetes clusters in Florida, 2007 and 2010.

Cluster	Population	Observed cases	Counties included^2^	No of Counties included	PR^1^	*p*-value
			**2007**			
Primary	1,239,259	123,822	9, 35, 69, 83, 97, 107	6	1.26	0.001
Secondary 1	127,334	9,992	5	1	1.5	0.001
Secondary 2	126,678	11,845	53	1	1.23	0.001
Secondary 3	65,436	5,874	87	1	1.28	0.001
Secondary 4	28,812	2,970	75	1	1.23	0.001
			**2010**			
Primary	218,337	30,829	111	1	1.44	0.001
Secondary 1	116,875	13,884	113	1	1.33	0.001
Secondary 2	38,127	6,790	39	1	1.39	0.001
Secondary 3	15,549	1,904	59	1	1.3	0.001

^1^Prevalence ratio.

^2^[5 = Bay 9 = Brevard 35 = Flagler 39 = Gadsden 53 = Hernando 59 = Holmes 69 = Lake 75 = Levy 83 = Marion 87 = Monroe 97 = Osceola 107 = Putnam 111 = St. Lucie 113 = Santa Rosa].

#### c) High DSME participation rates clusters

The spatial distribution of high DSME participation rate clusters (Fig 4) are consistent with the distribution of the rates in both 2007 and 2010 (Fig 2). There were 4 and 2 high participation rate spatial clusters of DSME participation rates in 2007 and 2010, respectively. The geographic sizes of the clusters identified in 2007 varied from one county (Secondary Cluster 2) to eight counties (Primary Cluster) (Table 2 and Fig 4).

These clusters were mainly located in the South-west, mid and mid-east part of Florida (Fig 4). The primary cluster was the largest in both geographic size (included 8 counties) and size of population in the cluster (2.1 million) and was located in mid-Florida. This cluster had a DSME participation rate that was 22% higher than the state average (Table 4). The single county cluster (Secondary Cluster 2) was composed of Leon county, which is an urban county that houses the state capital (Figs 1 and 4). This cluster had the highest DSME participation rate ratio (PRR = 1.56: p = 0.001) implying that this county had 56% higher participation rate than the state average. Although DSME participation rates increased from 2007 to 2010, fewer clusters were found in the western part of the panhandle and mid-east coast of Florida in 2010. However, DSME participation rate of the primary cluster in 2010 was almost equal (21% higher than the state average) to the rate of the primary cluster of 2007.

**Table 4:** Purely spatial clusters of High Diabetes Self-Management Education (DSME) program participation rates in Florida, 2007 and 2010.

Cluster	Population	Observed cases	Counties included in cluster^2^	No. of Counties included	PRR^1^	*p-value*
			**2007**			
Primary	2,052,527	128,477	9, 35, 53, 69, 105, 117, 119, 127	8	1.22	0.001
Secondary 1	854,415	57,913	15, 21, 71	3	1.29	0.001
Secondary 2	207,233	12,676	73	1	1.56	0.001
Secondary 3	311,922	13,961	85, 111	2	1.21	0.001
			**2010**			
Primary	1,606,390	138,762	9, 35, 61, 93, 105, 119, 127	7	1.21	0.001
Secondary 1	500,273	38,086	33, 91, 113	3	1.29	0.001

^1^Participation Rate Ratio.

^2^[9 = Brevard 15 = Charlotte 21 = Collier 33 = Escambia 35 = Flagler 53 = Hernando 61 = Indian River 69 = Lake 73 = Leon 85 = Martin 91 = Okaloosa 93 = Okeechobee 105 = Polk 111 = St. Lucie 113 = Santa Rosa 117 = Seminole 119 = Sumter 127 = Volusia].

Five Counties in mid-Florida (Sumter, Polk, Brevard, Volusia and Flagler) were consistently in high DSME participation rate clusters in 2007 and 2010 (Figs 1 and 4). Despite being a non-significant diabetes cluster county in 2007 and 2010, Sumter county was consistently part of a primary high DSME participation rate cluster in both 2007 and 2010 (Figs 1 and 4). Leon and several other counties of south-west Florida, which were significant clusters in 2007, became non-significant in 2010 (Figs 1 and 4).

#### d) Overlaps of diabetes prevalence and DSME participation rate clusters

The geographical locations of the clusters of high diabetes prevalence (both adjusted and unadjusted) rarely overlapped with those of high DSME participation rates. However, three significant cluster counties of high diabetes prevalence in 2007 (Hernando, Lake, and Flagler) were also significant high DSME participation rate clusters in 2007 (Figs 1 and 4). Similar overlaps were observed in Volusia, Polk, and Indian River counties in 2010 (Figs 1 and 4). While Leon county transitioned from not being part of a cluster (in 2007) to belonging to a high diabetes prevalence primary cluster (in 2010), the exact opposite happened in case of DSME participation rate since it transitioned from being part of a high DSME participation rate cluster (in 2007) to not being part of a cluster (in 2010).

### Predictors of disparities in DSME participation rates

Table 5 shows the univariable (unadjusted) associations of each of the predictors with DSME participation rate. Based on the multivariable model, significant predictors of DSME participation rates were proportion of rural population and proportion of Hispanic population (Table 6). There was also significant effect modification between the two variables. Thus, the relationship between the proportion of rural population and DSME participation rate depends on the proportion of Hispanic population and vice versa. There was no evidence of non-normality (p = 0.84) or heteroskedasticity (p = 0.49) of residuals of the OLS model. Additionally, both the robust Lagrange multiplier tests for lag (p = 0.22) and error (p = 0.33) showed no evidence of spatial dependence of the OLS residuals. There was also no evidence of multicollinearity since all VIF values were less than 10 (Table 6) and the multicollinearity condition number (6.15) was less than 20.

**Table 5:** Univariable associations between county characteristics and Diabetes Self-management Education (DSME) program participation rates in Florida, 2010.

Predictors	Coefficient (95% CI^1^)	*p*-value^2^
Proportion of having normal and less than normal weight	-0.033 (-0.405, 0.339)	0.86
Proportion of having overweight	0.308 (-0.221, 0.836)	0.25
Proportion of being obese	-0.092 (-0.421, 0.237)	0.58
Proportion of smokers	-0.229 (-0.562, 0.105)	0.18
Proportion of heavy drinkers	0.071 (-1.118, 1.259)	0.91
Proportion of doing exercise	0.219 (-0.197, 0.635)	0.30
Proportion of having overall good health	0.344 (-0.046, 0.734)	0.08
Proportion of having overall poor health	-0.344 (-0.734, 0.046)	0.08
Proportion of having limited walking capacity	-0.281 (-0.766, 0.204)	0.25
Proportion of having high school or less education	-0.239 (-0.415, -0.063)	0.01
Proportion of having some college education	0.267 (-0.236, 0.770)	0.29
Proportion of having college education	0.263 (0.063, 0.463)	0.01
Proportion of being white, non-Hispanic	0.037 (-0.169, 0.242)	0.72
Proportion of being black, non-Hispanic	0.099 (-0.216, 0.416)	0.53
Proportion of being other, non-Hispanic	0.595 (-0.340, 1.530)	0.21
Proportion of being Hispanic	-0.222 (-0.512, 0.069)	0.13
Proportion of being married	0.405 (-0.007, 0.817)	0.05
Proportion of being divorced/widowed/separated	-0.342 (-0.934, 0.250)	0.25
Proportion of being never married	-0.300 (-0.776, 0.176)	0.21
Proportion male	-0.527 (-0.938, -0.116)	0.01
Proportion female	0.527 (0.116, 0.938)	0.01
Proportion with age 18 to 44 years	-0.074 (-0.333, 0.186)	0.57
Proportion with age 45 to 64 years	0.066 (-0.235, 0.367)	0.66
Proportion with age equal or greater than 65 years	0.039 (-0.289, 0.367)	0.81
Median household income (in $10,000)	0.038 (0.007, 0.070)	0.02
Proportion of being unemployed	-0.168 (-1.344, 1.007)	0.78
Proportion of having diabetes	0.228 (-0.625, 1.081)	0.60
Proportion that take insulin	-0.030 (-0.294, 0.234)	0.82
Proportion that have diabetic complications (retinopathy)	-0.182 (-0.527, 0.163)	0.30
Proportion that have regular healthcare provider access	0.477 (0.083, 0.871)	0.02
Proportion of rural population	-0.086 (-0.153, -0.019)	0.01
Proportion that have insurance coverage	0.378 (0.108, 0.648)	0.01
Proportion with age ≥25 years with a college degree*	0.250 (0.058, 0.442)	0.01
Proportion below the federal poverty level*	-0.269 (-0.711, 0.173)	0.23

^1^Confidence interval.

^2^Potential statistical significance was assessed using a liberal critical p = 0.15.

**Table 6:** Results of ordinary least square regression model showing predictors of Diabetes Self-management Education (DSME) participation rates at the county level in Florida, 2010.

Predictor Variable	Coefficient (95% CI^1^)	SE^2^	t-value	p-value^3^	VIF^4^
Proportion of being Hispanic	-0.586 (-0.914, -0.259)	0.164	-3.58	0.0007	1.58
Proportion of rural population	-0.192 (-0.285, -0.010)	0.046	-4.15	0.0001	2.24
Proportion of Hispanic X Proportion of rural population Interaction	1.298 (0.191, 2.405)	0.554	2.34	0.02	2.12

^1^Confidence interval.

^2^Standard error.

^3^Statistical significance was assessed using a critical p = 0.05.

^4^Variance Inflation Factor.

### Predictors of clusters of high DSME participation rates

**Fig 5:**
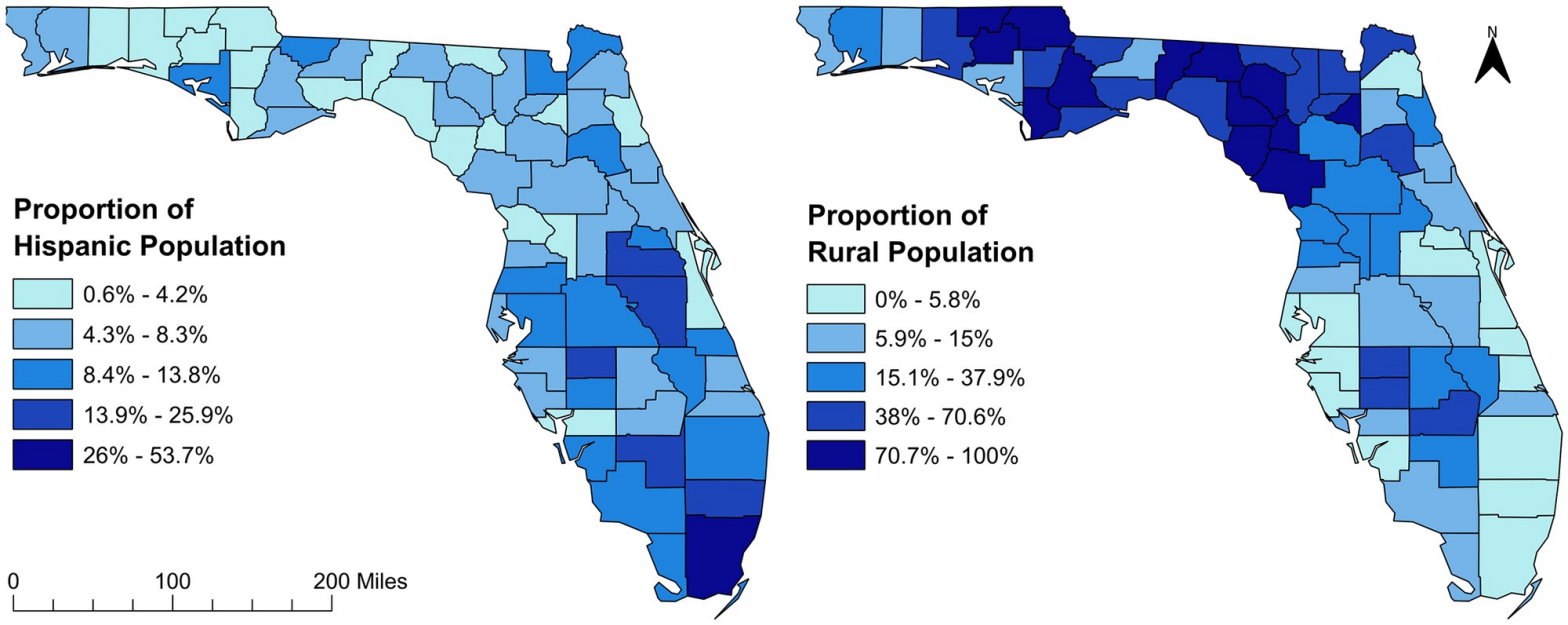
Distribution of significant predictors of diabetes self-management education program participation rates in Florida, 2010. Figure was created using the Free and Open Source Geographic Information System (GIS) software, QGIS. The basemaps used to create the maps were downloaded from the US Census Bureau Website: https://www.census.gov/geographies/mapping-files/time-series/geo/tiger-line-file.2010.html. The data on Hispanic population distribution can be downloaded from Florida Department of Health Website: http://www.floridahealth.gov/statistics-and-data/survey-data/behavioral-risk-factor-surveillance-system/index.html. The rural population data were downloaded from County Health Rankings Website available at: https://www.countyhealthrankings.org/app/florida/2021/measure/factors/58/data.

Table 7 shows variables that were considered as potential predictors of clusters of high DSME participation rates. In the final model, only the proportion of rural residents had significant association with clusters of DSME participation rate (Table 8). The geographic distribution of the significant predictors of DSME participation rates and clusters are shown in Fig 5. The proportion of Hispanic population showed a North-South gradient with the lowest proportions being observed in the north and highest in the South. In contrast, the proportion of rural population showed the reverse spatial trend with the lowest proportions of rural residents being observed in the South and highest in the north (Fig 5). Hosmer-Lemeshow goodness-of-fit test indicated no evidence of lack of fit (p = 0.58). Finally, the Moran’s I statistic showed no evidence of spatial dependence of the residuals (Moran’s I = 0.096; p = 0.15).

**Table 7:** Univariable associations between county characteristics and clusters of Diabetes Self-management Education (DSME) program participation rates in Florida, 2010.

Predictor variable	Coefficient (95% CI^1^)	*p*-value^2^
Proportion of having normal and less than normal weight	-4.047 (-15.049, 6.954)	0.471
Proportion of having overweight	6.959 (-10.260, 24.178)	0.428
Proportion of being obese	0.745 (-8.991, 10.481)	0.881
Proportion of smokers	2.448 (-8.106, 13.001)	0.649
Proportion of heavy drinkers	-8.540 (-44.742, 27.662)	0.644
Proportion of doing exercise	3.909 (-8.936, 16.754)	0.551
Proportion of having overall good health	8.364 (-5.580, 22.308)	0.240
Proportion of having overall poor health	-8.363 (-22.307, 5.580)	0.240
Proportion of having limited walking capacity	0.805 (-13.671, 15.280)	0.913
Proportion of having high school or less education	-3.862 (-9.764, 2.039)	0.200
Proportion of having some college education	10.378 (-5.524, 26.275)	0.201
Proportion of having college education	2.967 (-3.238, 9.172)	0.349
Proportion of being White, non-Hispanic	3.884 (-4.439, 12.207)	0.360
Proportion of being Black, non-Hispanic	-3.212 (-14.739, 8.316)	0.585
Proportion of being Other, non-Hispanic	-11.689 (-43.154, 19.776)	0.467
Proportion of being Hispanic	-3.575 (-15.880, 8.731)	0.569
Proportion of being married	13.310 (-0.592, 27.212)	0.061
Proportion of being divorced/widowed/separated	-4.752 (-23.354, 13.851)	0.617
Proportion of being never married	-15.613 (-33.068, 1.841)	0.080
Proportion male	-14.158 (-34.871, 6.556)	0.180
Proportion female	14.158 (-6.556, 34.871)	0.180
Proportion with age 18 to 44 years	-2.758 (-10.663, 5.146)	0.494
Proportion with age 45 to 64 years	1.023 (-7.915, 9.973)	0.822
Proportion with age equal or greater than 65 years	2.985 (-6.480, 12.449)	0.537
Median household income (in $10,000)	0.768 (-0.170, 1.706)	0.109
Proportion of being unemployed	21.916 (-14.617, 58.450)	0.240
Proportion of having diabetes	3.375 (-22.100, 28.761)	0.794
Proportion that take insulin	2.522 (-5.229, 10.274)	0.524
Proportion that have diabetic complications (retinopathy)	-3.372 (-14.733, 7.989)	0.561
Proportion that have regular healthcare provider access	2.344 (-10.635, 15.322)	0.723
Proportion of rural population	-3.696 (-7.140, -0.253)	0.035
Proportion that have insurance coverage	-4.383 (-4.835, 13.601)	0.351
Proportion with age ≥25 years with a college degree*	2.651 (-3.037, 8.602)	0.383
Proportion below the federal poverty level*	-13.309 (-29.933, 3.314)	0.117

^1^Confidence interval.

^2^Potential statistical significance was assessed using a liberal critical p = 0.15.

**Table 8:** Results of the final logistic model showing statistically significant predictor of clusters of Diabetes Self-management Education (DSME) participation rates at the county level in Florida, 2010.

Predictor variable	Odds Ratio	Coefficient (95% CI^1^)	SE^2^	Wald Chi-Square	*p*-value^3^
Proportion of rural population	0.025	-3.696 (-7.140, -0.253)	1.757	4.426	0.035

^1^Confidence interval.

^2^Standard error.

^3^Statistical significance was assessed using a critical p = 0.05.

## Discussion

This study investigated geographic disparities of diabetes prevalence and Diabetes Self-management Education (DSME) Program participation rates in Florida. Some previous studies have shown evidence of geographic disparities in both the burden of diabetes and access to healthcare for individuals with diabetes in the United States [15,19,39–42]. One of the ways that the CDC is trying to address these disparities is by providing diabetes preventive programs (DPP). The DSME, which is run by the American Diabetes Association (ADA) designated centers, is one of the programs intended to educate diabetic patients on disease management. While DPP aim at reducing diabetes incidence in prediabetic populations, DSME targets to reduce diabetes related complications in diabetic populations [43]. However, DSME participation rates across the states are considerably low [44]. In addition, DSME centers are not geographically distributed equitably resulting in potential disparities in DSME participation rates [45,46]. Although disparities in DSME program availability have been investigated [16], no previous studies have investigated disparities of DSME participation rates and yet this information is critical for guiding resource allocation for DSME program implementation. The findings of the current study help to fill this gap and are useful in guiding evidence-based health planning and resource allocation in combating the diabetes problem.

Diabetes clusters identified in the north and central parts of Florida are consistent with findings by Barker *et al*., who reported that several counties of northern Florida were a part of the diabetes belt, an area of the southeast US where diabetes prevalence was significantly higher than the rest of the country [19]. This is probably due to geographical differences in the distribution of socio-cultural and genetic factors [19]. However, clusters of high diabetes prevalence identified in central Florida in the current study were not included in the diabetes belt of the study by Barker *et al*. This may be due to the fact that the study by Barker *et al*. used an arbitrary cut-off value of diabetes prevalence to define the diabetes belt [19]. Their study defined counties with diabetes prevalence of ≥11% as belonging to the diabetes belt whereas our study has used a rigorous statistical approach to identify high prevalence diabetes clusters. Patterns of diabetes distribution similar to those of the current study were reported in another study which also identified several socioeconomic determinants (high levels of poverty, percentage of non-Hispanic black, obesity and physical inactivity) as significant predictors of the reported hotspots of diabetes prevalence in northern Florida [39].

Although high diabetes prevalence clusters were observed both in the northern and central parts of Florida, high DSME participation rate clusters were only observed in central Florida. It was concerning that seven counties in northern and central Florida had significant increases in diabetes prevalence during the study period and yet they had significant decreases in DSME participation rates during the same time period. This might be due to lack of DSME program facilities in the rural counties of northern Florida (Paul et al., 2018). This is supported by the findings of the OLS model used to investigate predictors of DSME participation rates which revealed that rural counties and those with a higher proportion of Hispanic population tended to have lower DSME participation rates. The findings of the logistic model investigating the predictors of a county being in a high DSME participation rate cluster almost mirrored those of the OLS model. The odds of a county being in a DSME high participation rate cluster was significantly lower for counties with higher percentages of rural residents compared to those with lower percentages of rural residents. In fact, the largest cluster of high DSME participation rates in 2010 was located in mid-east Florida where almost all of the counties were urban. These rural areas with high diabetes prevalence, but low DSME participation, are of significant concern as these areas could possibly contribute the most in economic burden of diabetes having a large diabetic population with more diabetes related complications.

The observed low DSME participation rates in counties with higher proportions of rural populations may be due to the lack of available DSME programs. There is evidence that rurality influences access to DSME more than socio-economic status such as poverty level [47]. Thus, it is possible that rural areas of Florida have fewer DSME centers despite having a high burden of diabetes. This is consistent with the findings of a study by Paul *et al*., which reported that southeast regions of the US, including rural northern Florida, had higher diabetes prevalence but fewer DSME centers [45]. Suffice it to say that despite having high prevalence of diabetes, Florida has inequities in distribution of DSME programs. Another study reported that almost two-thirds of rural counties of the US did not have a single DSME program [46].

The DSME participation may not depend on DSME program availability only. A study by Rutledge and co-workers reported that higher odds of having DSME centers (program availability) were associated with high percentage of diabetic and insured population, low percentage of population with high school education or less, and low unemployment rate [46]. These factors could have also explained DSME participation if DSME program availability was the principal determinant of DSME participation rate. In the current study, although the proportion of insured population and those with high school education or less had significant univariable positive and negative associations with DSME participation rates, respectively, they were not significant in the final model. The reason for this is unclear but might be due to the fact that some other factors (e.g. availability of transport to DSME centers especially in rural areas) might be more important determinants of the DSME participation in Florida [46,48,49]. Unfortunately, we did not have access to transportation data and therefore could not investigate this factor. Although counties with higher proportion of diabetic population are more likely to have DSME centers [16], the current study shows that DSME participation rate did not depend on whether a county has higher or lower proportion of diabetic population. This again implies that even if DSME programs are available in rural Florida which had high diabetes prevalence, participation rate could be lower. It has been reported that health related program participation often depends on behavioral factors e.g., awareness and willingness to participate [49]. A New Jersey study also reported that DSME participation in certain counties did not always reflect DSME program availability [50]. Thus, DSME participation is affected by not only program availability but also by acceptability, accessibility and other factors [51,52]. The implication of this is that the low DSME participation rates observed in rural areas of Florida could be the result of complex interactions between cultural, psychological, environmental, economic, and human resource factors such as transportation [46,48], lack of specialists in rural areas, lack of diabetes educators [53], participants’ literacy level, language barriers [54], lack of time, lack of childcare, participants’ shame of illness, and participants lacking interest in their health [49].

The significant negative association between county level DSME participation rates and proportion of Hispanic population suggests that racial disparities play a significant role in geographic disparities of DSME participation rates. Previous studies showed that Hispanics, Blacks, and Asians had low healthcare access compared to Whites with Hispanics facing the greatest barrier [55]. An individual level study reported that language barrier could significantly influence DSME participation [49,56]. In the United States, patients who attend DSME programs tend to be Caucasian and English-speaking [56]. Although other studies have reported that counties with higher non-Hispanic Black population tended to have higher diabetes prevalence in Florida [15], there was no association between percentage of non-Hispanic black population and DSME participation rates in the current study. Rather, DSME participation rates in the current study tended to be lower in the southern Florida counties that had higher proportion of Hispanic population but lower diabetes prevalence. Thus, DSME participation rates in Florida did not depend on the burden of diabetes but might be more influenced by racial distribution. At the individual level, low DSME participation among Hispanics have been attributed to financial constraints, work schedule conflicts, and lack of transportation [57–59]. Hence, family support, positive relationships with health care providers, and group support from DSME classes have been shown to increase DSME participation among Hispanics [57,60].

### Strength and weakness

This is the first study investigating geographic disparities of DSME participation rates in Florida using rigorous statistical approaches. Understanding the relationship between diabetes burden and DSME participation rates and identifying areas with high diabetes burden but low DSME participation areas is crucial for guiding planning to reduce disparities in access to care for diabetic patients and improve DSME participation rates. However, this study is not without limitations. Data on diabetes and DSME participation were self-reported and so may be prone to reporting bias. The BRFSS does not categorize diabetes as either type 1 or type 2 and so this differentiation could not be made. However, 90–95% of all diabetes cases in the United States are type 2 diabetes [44]. These limitations notwithstanding, the findings of this study provide useful information to guide health planning to reduce disparities in diabetes burden and DSME participation rates.

## Conclusions

This study confirms geographic disparities of diabetes prevalence and DSME participation rates. It also identifies areas that have high diabetes prevalence but low DSME participation rates. These areas are of concern and will need specific attention in order to address the issue of disparities in healthcare access for diabetic patients in Florida. The study has also demonstrated the usefulness of GIS and spatial epidemiologic/statistical approaches in investigating disparities in diabetes burden and DSME participation rates. Study findings are useful for guiding resource allocation geared towards reducing disparities and diabetes burden in Florida.
